# Distinctive Frontal and Occipitotemporal Surface Features in Neglectful Parenting

**DOI:** 10.3390/brainsci11030387

**Published:** 2021-03-18

**Authors:** Inmaculada León, María José Rodrigo, Ileana Quiñones, Juan Andrés Hernández-Cabrera, Lorna García-Pentón

**Affiliations:** 1 Instituto Universitario de Neurociencia, Universidad de La Laguna, 38200 La Laguna, Spain; mjrodri@ull.es (M.J.R.); jhernand@ull.edu.es (J.A.H.-C.); 2 Facultad de Psicología, Universidad de La Laguna, 38200 La Laguna, Spain; 3 Basque Center on Cognition, Brain, and Language, 20009 Donostia-San Sebastián, Spain; i.quinones@bcbl.eu; 4 MRC Cognition & Brain Sciences Unit, University of Cambridge, Cambridge CB2 7EF, UK; lg620@cam.ac.uk

**Keywords:** neglectful parenting, cortical thickness, surface area, alexithymia, mother-child interaction

## Abstract

Although the brain signatures of adaptive human parenting are well documented, the cortical features associated with maladaptive caregiving are underexplored. We investigated whether cortical thickness and surface area vary in a small group of mothers who had neglected their children (24 in the neglect group, NG) compared to a control group of mothers with non-neglectful caregiving (21 in the control group, CG). We also tested whether the cortical differences were related to dyadic mother-child emotional availability (EA) in a play task with their children and whether alexithymia involving low emotional awareness that characterizes the NG could play a role in the cortical-EA associations. Whole-brain analysis of the cortical mantle identified reduced cortical thickness in the right rostral middle frontal gyrus and an increased surface area in the right lingual and lateral occipital cortices for the NG with respect to the CG. Follow-up path analysis showed direct effects of the right rostral middle frontal gyrus (RMFG) on the emotional availability (EA) and on the difficulty to identify feelings (alexithymia factor), with a marginal indirect RMFG-EA effect through this factor. These preliminary findings extend existing work by implicating differences in cortical features associated with neglectful parenting and relevant to mother-child interactive bonding.

## 1. Introduction

Although most parents can respond appropriately to their child’s needs, extreme cases of insensitive caregiving do exist. In fact, neglect is the most common form of child maltreatment, which involves a drastic failure to provide the child with food, clothing, shelter, medical care, supervision, or emotional support that places the child’s safety at risk [1,2]. Being severely neglected entails negative behavioral, neurobiological, and clinical consequences for the child [3,4]. Although the brain signatures of adaptive parenting are well documented (e.g., [5]), the neural mechanisms associated with neglectful caregiving are comparatively less studied. Nevertheless, identifying the cortical features linked to neglectful behavior could be relevant to better understanding the cycle of maladaptive parenting.

To this end, this study seeks to advance work on maladaptive parenting by investigating whether cortical features vary in mothers who neglect their children. Specifically, we address gaps in knowledge in two directions. First, we identify differences in cortical thickness and surface area between a group of those mothers who had neglected their children, a neglect group (NG), and a control group of mothers (CG) with non-neglectful caregiving. Second, we examine to what extent those cortical features potentially associated with the NG were related to the quality of a mother-child interactive play task (cortical–emotional availability associations), and we determine whether alexithymia could play a role in these associations.

### 1.1. Neuroanatomical Correlates of Neglectful Caregiving

Previous neuroimaging research on mothers showing neglectful caregiving has identified functional and structural variations mostly in frontal, cingulate, and occipitotemporal areas. In a functional magnetic resonance image (fMRI) study [6], mothers who neglected their children versus control mothers showed reduced activations in response to infant and adult crying faces, both in a frontal cortical area (inferior frontal gyrus) as well as occipitotemporal cortical areas (lingual and fusiform). In the same study, mothers with neglectful behavior also showed an attenuated activation to infant crying faces in left middle frontal and anterior cingulate areas, underscoring their specific difficulties with responding to the infant’s emotional cues. In a voxel-based morphometric study [7], grey matter volume reductions in the right insula, anterior/middle cingulate, and inferior/middle frontal gyrus, and greater volume in the right fusiform and cerebellum, were obtained for mothers with neglectful behavior as compared to control mothers.

Neuroimaging research has also documented the critical role of regions such as the medial and lateral prefrontal cortex (PFC) and anterior cingulate cortex (ACC) in emotion regulation [8,9]. Emotion regulatory capacities are vital for supporting parents’ ability to be perceptive, responsive, and flexible in the face of challenging child behavior [10,11]. Likewise, occipitotemporal visual areas specialized in the processing of faces are crucially involved in sensitive parenting [5,12]. Moreover, these areas have shown their involvement in the altered processing of a child’s crying in mothers who neglect their children, affecting the caregiver’s ability to be attuned to the infant’s needs [6,13]. However, less is known about the potential role of cortical thinning and surface area in the specific neural underpinnings of both emotion regulation and face-responsive areas in the context of neglectful caregiving.

Cortical thickness (CT) and cortical surface area (SA) are morphological features of the cerebral cortex that follow different genetic, phylogenetic, and developmental routes, capturing the anatomical and functional variability in the brain [14]. Studies in normal mothering have shown that the brain undergoes changes in cortical thickness, with differences observed before and after pregnancy [15,16] and in primiparous mothers versus non-mothers [17]. There is also evidence of cortical variations in the maternal brain in a group of first-time mothers, showing that a later postpartum month was associated with increased cortical thickness in the prefrontal cortex, the lateral occipital areas, and the fusiform gyri [18], suggesting brain structural adaptations to the child’s evolving needs. From a complementary perspective that compares extreme variations in maternal caregiving, the current study examined possible differences in cortical features (CT and SA) in those mothers who had neglected their child versus control mothers, and whether these cortical differences are related to observed mother-child interactive behavior.

### 1.2. Relations between Brain Features, Personality Traits and Quality of Mother-Child Interaction

As for the second direction taken in this study, we favored using an observational mother-child regulatory task over self-report instruments informed by the mothers. We used emotional availability (EA), measured through a mother-childfree play task, as a proxy for the quality of mother-child interactions [19,20]. This measure is predictive of the mother’s reported child attachment [21]. EA refers to sensitive and responsive dyadic exchanges and the cognitive organization of coordinated mother-child actions towards achieving joint goals. Mother-child synchrony in daily exchanges is a crucial feature in this task that is related to the infant’s attachment quality [22,23]. Moreover, results on this task discriminated the low performance in the emotional availability of neglectful versus non-neglectful dyads very well [7]. Low performance in EA was also related to reductions in the structural connectivity of white matter tracts interconnecting the face-responsive cortex with the limbic and frontal areas in mothers with neglectful behavior [24].

Some differences in trait alexithymia found in neglectful versus non-neglectful caregiving [25] could play a role in the cortical differences—EA associations. Alexithymia is defined as metacognitive impairments in emotional awareness that involve difficulties in identifying and describing one’s emotions and minimizing emotional experience by focusing attention externally [26,27,28], which may affect the mother-child exchanges. Brain regions hypothesized as featuring NG and CG differences have been described as part of alexithymia neural correlates. High compared to low alexithymic women showed reduced activations in the orbitofrontal cortex (OFC) and ACC while empathizing with an imagined young child [29]. Moreover, recent evidence has shown associations between cortical thickness features and personality traits [30,31]. Taking this evidence into account, we have started from a model that considers variations in cortical features as antecedents, presumably related to differences in alexithymia and the performance in the mother-child play task. Finally, since mothers with neglectful behavior have shown a history of childhood maltreatment and life stress that usually entails early brain alterations [1,4], it is important to consider its possible effect on cortical features.

In sum, this study addresses the lack of evidence in neuroimaging research on possible differences in the cortical features in regions associated with neglectful versus non-neglectful caregiving. This study also tries to find evidence of the relationships of those differential cortical features with the quality of mother-child interactions. Following results obtained with other neuroimaging measures in mothers with neglectful caregiving reported here, we expected cortical differences in both frontal and occipitotemporal areas. We also tentatively expected that alexithymia as an emotional personality trait would contribute to the association between those cortical features and observed dyadic emotional availability.

## 2. Materials and Methods

### 2.1. Participants

Forty-five mothers (24 NG and 21 CG) of the forty-seven scanned samples participated in the experiment. They were all recruited through the same Primary Health Center. All subjects gave their written informed consent for inclusion before they participated in the study. The study was conducted following the Declaration of Helsinki, and the protocol was approved by the Ethics Committee of the University of La Laguna (code: CEIBA2017-0254; date of approval: 25 July 2017). General inclusion criteria were being the biological mother of a child under three years old who had not been placed in foster care at any point in their history, nor been born prematurely or suffered perinatal or postnatal medical complications, according to the pediatricians’ reports. Specific inclusion criteria for a mother in the neglect group were a substantiated case of neglect registered in the last 12 months by Child Protective Services (CPS) and complying with the indicators of the Maltreatment Classification System (MCS) for severe neglect [32]. The control group’s inclusion criteria were having negative scores in all the MCS neglect indicators and the absence of CPS or Preventive Services records for the family.

As for the mothers’ sociodemographic profile, they were all in their early 30s, had more children and a similar mean age of the target child, lived in rural areas, and shared a similar low level of education; mothers in the NG were more likely than those in the CG to live in single-parent families and to receive financial assistance (Table 1). According to the neglect risk profile rated by the social workers, most mothers in the neglect group had a history of childhood abuse or neglect and scored positively in neglectful caregiving indicators (see Supplementary S1: Participant recruitment strategy and procedure; Supplementary S2: Risk profile measures).

### 2.2. MRI Data Acquisition

High-resolution T1-weighted Magnetisation Prepared Rapid Gradient Echo or MPRAGE anatomical volumes were acquired on a General Electric 3T scanner located at the University Hospital’s Magnetic Resonance Service for Biomedical Research at the University of La Laguna. A total of 196 contiguous 1 mm sagittal slices were acquired with the following parameters: repetition time = 8.716 ms, echo time = 1.736 ms, field of view = 256 × 256 mm^2^, in-plane resolution = 1 mm × 1 mm, flip angle = 12.

During the ongoing data acquisition, the T1-MRI was screened for clear artifacts by the MRI technician. Participants were rescanned on the way if an obvious artifact was detected. If the technician (or the trained experimenter) detects any potential alteration clinically relevant, the images were passed to the neuroradiologist for further examination. All the participants signed in the consent form to agree to receive information if any alteration was detected. No data were rejected at this stage.

### 2.3. Cortical Thickness and Surface Area

After data conversion into Nifti format, the trained experimenter visually checked the quality of each T1-MRI image using the MRIcron software. The experimenter thoroughly inspected the images, taking into account these main criteria: motion artifacts (ghosting or blurring effects in the images due to mainly participant’s gross movements, breathing, cardiac effects, etc.), ringing artifacts (truncation/Gibbs artifacts) and susceptibility artifacts (image distortions due to inhomogeneities of the magnetic field). No data were rejected, followed visual inspection, and all passed into Freesurfer’s automated preprocessing pipeline (recon-all). However, data from two control participants had to be excluded due to FreeSurfer cortical reconstruction process (recon-all) failures. Freesurfer’s outputs from all participants were also visually inspected, as explained below. No statistical extreme outliers were identified below or above 2.5 SD for the extracted cluster brain measures that may have driven the results obtained.

Cortical thickness (CT) and surface area (SA) measures were obtained using FreeSurfer (version 5.1) (http://surfer.nmr.mgh.harvard.edu/, accessed on 10 February 2020). Surface cortical reconstruction included: motion correction, skull tripped, automated transformation into Talairach space, subcortical white matter, deep gray matter volumetric structure segmentation, triangular tessellation of the white surface (corresponding to the grey/white matter boundary), and pial surface (corresponding to the pia mater), and automated topology correction and surface deformation that optimally place the (inner) white surface and the (outer) pial surface [33,34]. Some deformations included surface inflation and a high-dimensional nonlinear registration to a spherical atlas. The segmentation and deformation algorithms produce representations of the CT that is the average of the closest distance from the white to the pial surface and from the pial to the white surface at each vertex on the tessellated surfaces. The SA is computed at the white surface, which is less sensitive to cortical thickness variations, and measured at each vertex as one-third of the area of each triangle that meets the vertex; in other words, it is the sum of the area of all the triangles that meet the vertex divided by three [14]. The CT and SA maps were smoothed using a 15 mm with a full-width at half maximum (FWHM) Gaussian filter.

For the quality check of FreeSurfer’s outputs, the individual white and pial surfaces were displayed in Freeview to inspect visually if they accurately followed the grey and white matter boundaries. No errors were observed. FreeSurfer also provided an averaged brain (fsaverage) in Montreal Neurological Institute (MNI) standard space that was used to compare and visualize results.

### 2.4. Behavioral and Personality Measures

The Mini-International Neuropsychiatric Interview (M.I.N.I. 6.0), in the Spanish version [35], including 15 major psychiatric disorders, was administered (see Supplementary Table S1 Psychopathological conditions stratified by Group). The score obtained for each disorder corresponds to a cumulative scoring of symptoms and not to a categorical cut-off classification. The two groups mainly differed in five psychopathological variables, marked in italics, which survived the Bonferroni correction and were submitted to a Principal Component Analysis. Results gave one-factor solution: “Psychiatric Disorders” (PD), with moderate inter-correlations among the five variables, Kaiser-Meyer-Olkin (KMO) test = 0.65, Eigenvalue = 2.53, with an explained variance of 51%, with the coefficient scores in PD being higher in the NG (see Table 1). None of the mothers in either group were being medicated for psychiatric disorders at the time of testing. The coefficient score in PD was used as a regressor in the FreeSurfer model to control as much as possible for its effect on cortical differences.

Dyadic Emotional Availability was measured in the context of mother-childfree play using the Emotional Availability Scale (EA): Infancy to Early Childhood Version [36]. EA signifies the quality of emotional exchanges, focusing on partners’ accessibility to each other and their ability to read and respond appropriately to each other’s communications. Two external observers blind to the mothers’ groupings made the videos’ ratings, and the inter-rater reliability of the ratings in each scale (Kappa score, 0 to 1) was calculated. The scale operationalizes four aspects of parental behavior: *Sensitivity* (the parent shows contingent responsiveness to child signals and demands, *K* = 0.94); *Structuring* (the parent appropriately facilitates the child’s play, *K* = 0.90); *Non-intrusiveness* (the parent can support the child’s play without being over directive and/or interfering, *K* = 0.87); *Non-hostility* (the parent can behave with the child in a way that is not rejecting or antagonistic, *K* = 0.92). The scale also measures two aspects of child behavior: *Responsiveness* (the child’s ability and interest in exploring on his or her own and in responding to the parent’s bids, *K* = 0.92), and *Involvement* (the child’s ability and willingness to engage the parent in interaction, *K* = 0.86). To obtain a simpler structure of the six standardized scales, a Principal Component Analysis was performed (see Supplementary Table S2: Inter-rater reliabilities and one-factor component loadings of the Emotional Availability Scales). The result yielded a single-factor structure given the existence of high inter-correlations between the variables in the mother-child dyad: *KMO* = 0.84, *Eigenvalue* = 4.49, with an explained variance of 75%. The coefficient score in this single factor was used as a measure of dyadic emotional availability.

Finally, the Toronto Alexithymia Scale (TAS-20) [37,38] measures the difficulty in identifying and expressing one’s emotions. The 20 items are scored on a 5-point scale from strongly disagree to strongly agree. The TAS-20 has a three-factor structure: Factor 1, Difficulty in Describing Feelings (DDF; *α* = 0.73); Factor 2, Difficulty in Identifying Feelings (DIF; *α* = 0.90); and Factor 3, Externally Oriented Thinking (EOT; *α* = 0.50). The overall score averaging the three-factor scores was also calculated.

Statistical analyses comparing measures between the two groups showed significantly higher alexithymia and lower dyadic emotional availability in the NG than in the CG (see Table 2).

### 2.5. Statistical Analyses for Brain Measures

As no prior research has examined differential cortical features in neglectful caregiving, we probed the entire cortical mantle with an exploratory whole-brain analytic approach to allow the discovery of a full range of cortical differences associated with neglectful versus non-neglectful caregiving. An analysis of covariance (ANCOVA) was performed in FreeSurfer to explore regional differences in CT and SA between the NG and CG, adjusted for age. The model also included one regressor described above as Psychiatric Disorders, once the potential collinearity of this psychiatric condition with the group was ruled out (see Supplementary Table S3: Collinearity indexes between the Group and the psychopathological conditions). Total intracranial volume (TIV) was also included as a nuisance covariate in the SA analysis.

Smoothed Gaussian Monte Carlo (MCZ) simulation (10,000 iterations) was used to perform cluster-wise (CW) correction of multiple comparisons, with an initial vertexwise cluster-formation threshold (CFT) ≤ 0.001 [39]. However, according to these authors, if no cluster is found, a less strict CFT (≤0.005) can be used for cortical thickness (CT) analyses. At this threshold, CT is less affected by false-positive rates (FPR) than Surface Area (SA) analyses if the FWHM > 10 mm is fulfilled, as is our case (FWHM = 15 mm). To further minimize FPR, we checked whether the clusters surviving the former criteria are also hypothesis-driven, corresponding to the regions (e.g., frontal and occipitotemporal) mostly found in the reported studies. Cluster formation was performed in each hemisphere separately and then corrected for both hemispheres using Bonferroni correction at *p*-value < 0.05. Regional differences are described based on the FreeSurfer Desikan/Killiany parcellation atlas [40].

### 2.6. Statistical Analyses for Cortical-EA Associations

Path analyses were planned to test the potential relation between cortical features (CT/SA), alexithymia (assuming that it differs between groups), and emotional availability (EA) in the whole sample. According to the starting model explained above, the cortical measure/s acted as the exogenous variable/s, the alexithymia overall factor score and/or specific factors acted as the variable/s between brain measures and EA, and EA acted as the completely endogenous outcome variable. The brain measures’ values were the mean thickness and surface area extracted for the differential clusters in the above analyses. The alexithymia scores most suited to enter in the path analysis were those of the averaged factor and/or the three specific factors, provided that they correlated with both the cluster values and the EA scores.

Since most mothers in the NG (16 out of 24) had suffered childhood maltreatment (neglect or physical abuse), this condition should also be considered. Previous studies showed that a history of child abuse was related to cortical volumetric alterations [41] and poor quality of mother-infant interactions [42]. Based on this evidence, path analyses were also planned where the mother’s own childhood maltreatment acted as the exogenous variable, given its condition as temporal antecedent. Cortical measures acted as the variables between maltreatment condition and EA, and EA acted as the completely endogenous outcome variable. All the analyses were performed with R Core team [43] and Lavaan R [44] packages.

## 3. Results

### 3.1. Differential Cortical Features in Neglect and Control Groups

**Figure 1:**
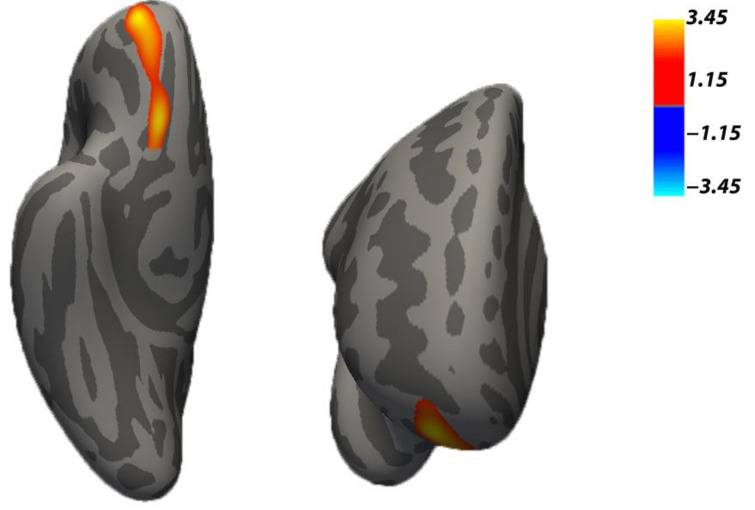
The cortical thickness reduction in Neglect versus Control groups. The cluster comprises the right rostral middle frontal gyrus, as part of the dorsolateral frontal gyrus, and the lateral and medial orbitofrontal areas. Background brain images represent the inferior (**left** image) and anterior (**right** image) views of the right inflated hemisphere of (FreeSurfer) fsaverage brain. Note: *p* < 0.05 cluster-wise corrected, 10,000 Monte Carlo iterations, age, and Psychiatric Disorders were included as nuisance covariates for the cortical thickness analysis.

The results in CT with a CFT = 0.001 showed no cluster formation. Then, a CFT = 0.005, also permitted for CT with a FWHM >10 mm, was applied, resulting in one cluster with a pattern of cortical grey matter reduction in mothers of the NG compared to the CG (CG > NG). The cluster (*p* = 0.014, Table 3 upper and Figure 1) spanned several contiguous anatomical regions in the right hemisphere, involving the right rostral middle frontal gyrus (RMFG) and the lateral and medial orbitofrontal cortex (OFC), which corresponds to the frontal regions previously hypothesized. The structural MRI data and the covariates scores that support the findings of this study are available in a G-Node Infrastructure (GIN) repository (for details, see the Data Availability statement at the end of the paper).

**Figure 2:**
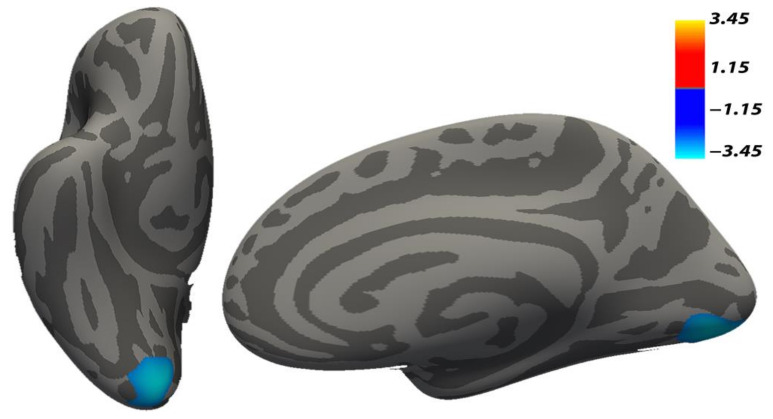
The increased cortical surface area in Neglect versus Control groups. The cluster comprises lingual and lateral occipital regions. Background brain images represent the inferior (**left** image) and medial (**right** image) views of the right inflated hemisphere (FreeSurfer) fsaverage brain. Note: *p* < 0.05 cluster-wise corrected, 10,000 Monte Carlo iterations, age, total intracranial volume (TIV) and Psychiatric Disorders were used as nuisance covariates for the cortical surface area analysis.

The results in the cortical surface area, applying a CFT = 0.001, revealed a significant pattern of greater surface area in mothers of the NG with respect to the CG (CG < NG) in the occipitotemporal region previously hypothesized. The analysis showed a large cluster (*p* = 0.0002, see Table 3 bottom and Figure 2) extending over the right lingual and lateral occipital gyri of the right hemisphere.

### 3.2. Role of Alexithymia in Cortical-EA Associations

**Figure 3:**
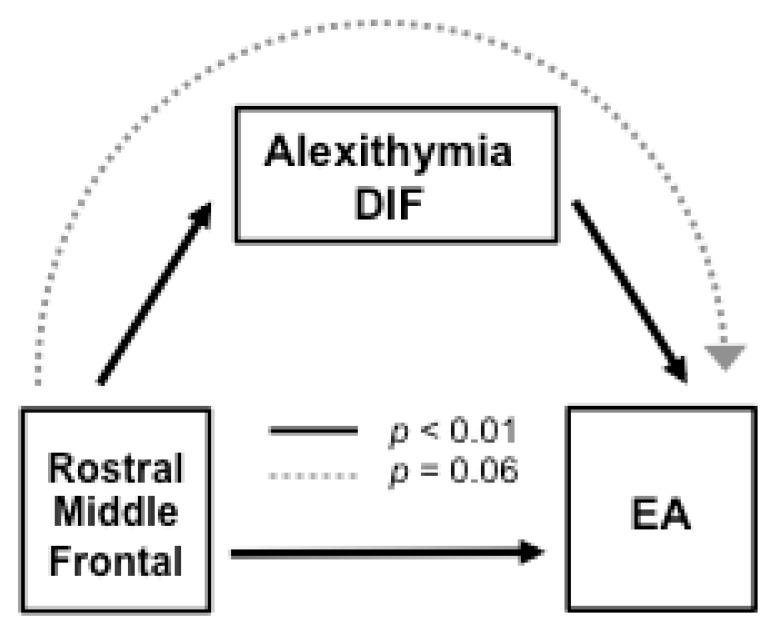
Path analysis showing cortical brain associations with alexithymia Difficulty Identifying Feelings (DIF) and emotional availability (EA) in the whole sample. The model shows the direct effects of the right rostral middle frontal gyrus (RMFG) on alexithymia DIF, and on the emotional availability (EA) exhibited in the mother-child interactive play task. The indirect effect through alexithymia DIF was marginal, but the total effect of RMFG thickness on EA remained significant.

As predicted, both the average score of alexithymia and its three factors were higher for the NG than for the CG (see Table 2). However, only the factor Difficulty in Identifying Feelings (DIF) correlated both with the right rostral middle frontal gyrus (RMFG) cortical thickness (positively, *r* = 0.33, *p* = 0.05) and with Emotional Availability (negatively, *r* = −0.30, *p* = 0.05). Therefore, DIF was considered to be a good candidate for testing its role in cortical-EA associations. A path analysis was performed to test the role of DIF in the relationship between the cortical thickness scores in the middle frontal gyrus (RMFG) and Emotional Availability (EA) performance. All standard errors and hence the values of the z-statistic have been estimated from 1000 bootstrap samples. The results are depicted in Figure 3, showing that the alexithymia factor Difficulty Identifying Feelings (DIF) was negatively related to EA scores (*B* = −0.378, *z* = −2.829, *p* = 0.005). The path analysis also revealed significant direct positive effects of RMFG thickness on alexithymia DIF (*B* = 1.982, *z* = 2.761, *p* = 0.006), and on EA scores (*B* = 1.795, *z* = 2.846, *p* = 0.004). A marginal indirect negative effect of RMFG thickness on EA through alexithymia DIF was also found (*B* = −0.749, *z* = −1.850, *p* = 0.064). However, the total positive effect of RMFG thickness on EA, once the indirect effect through alexithymia DIF had been subtracted, remained significant (*B* = 3.400, *z* = 3.508, *p* = 0.000). Given that the number of estimated parameters is equal to the number of different information units in the covariance matrix, no goodness of fit was obtained (*X*^2^ and degrees of freedom are equal to zero).

The second set of path analyses tested the extent to which the childhood maltreatment condition was related to the cortical measures (RMFG and Occipital Cluster) and EA scores. The maltreatment condition showed a direct and significant negative effect on EA scores (*B* = −1007, *z* = −3.181, *p* = 0.001). However, no significant effects of the maltreatment condition on the two cortical measures were found (*p* = 0.586 for RMFG and *p* = 0.190 for Occipitotemporal Cluster), and the cortical measures on EA (*p* = 0.074, and *p* = 0.935, respectively). No significant indirect effects of the maltreatment condition on EA through cortical measures were found (*p* = 0.664, and *p* = 0.947, respectively). The goodness of fit was adequate: *X^2^* = 0.375, *p* = 0.05, Normed Fit Index (NFI) = 0.978, Comparative Fit Index (CFI) = 1, Root Mean Square Error Approximation (RMSEA) = 0. Consequently, suffering childhood maltreatment was greatly associated with poor dyadic performance, with this direct relationship explaining all the variance, without the contribution of any other effects associated with brain variables.

## 4. Discussion

This study investigated differential brain morphometric patterns in the NG and CG, and their potential association with the mother-child interactive task, adding evidence for brain structure-emotional availability associations. We found preliminary evidence of differences in the cortical features between mothers who had neglected their children and control mothers using surface-based analysis for the first time in the study of this population. We also found that these cortical differences and trait alexithymia that influence mothers’ capacity to identify their emotions effectively were associated with emotional availability during mother-child exchanges.

Mothers with neglectful behavior compared to mothers with non-neglectful behavior showed cortical thickness reduction in the right rostral middle frontal gyrus and orbitofrontal cortex (RMFG/OFC). Both areas are involved in the frontal-limbic modulation of cognitive control and affective processing [45,46]. Adults exposed to childhood maltreatment, which is also the case in mothers with neglectful caregiving, had less cortical thickness in the lateral orbitofrontal cortex (OFC) and the pericalcarine cortex related to the perpetration of physical aggression [47]. Control-related regulatory capacities are especially critical for mothers dealing with adverse circumstances (e.g., childhood maltreatment, poverty, one-parent household, unemployment) since lower capacities put them at greater risk for maltreating their children [48].

In turn, evidence of greater cortical surface area in the NG versus CG was found in a region comprising lingual and lateral occipital cortices. The lateral occipital region is bi-directionally connected to the fusiform and inferior occipital areas forming the face-responsive network [49,50], crucially involved in sensitive parenting [5,12]. Occipitotemporal regions are known to be under-activated in the emotional processing of infant and crying faces in neglectful caregiving, affecting the caregiver’s ability to be attuned to the infant’s needs [6]. Both frontal and occipitotemporal group effects support our hypothesis based on previous neuroimaging studies with this population. Gray matter volume reductions in frontal regions and greater volume in the fusiform area were associated with neglectful mothering [7]. The frontal and fusiform areas were found to undergo critical morphometric adaptations indexed by cortical thickness increases in the postpartum period in a normal range of sensitive mothering [18]. In sum, evidence from several sources highlights the relevance of control and face-responsive areas to support variations in the quality of maternal caregiving.

Interestingly, the reverse pattern of greater surface area/greater volume in occipital regions and reductions in cortical thickness/gray matter volume in the frontal region is in line with the differential pattern obtained in the oscillatory rhythms of the NG in response to emotional stimuli when compared to the CG [51]. The higher the theta band oscillations (and lower alpha) at occipital sites, the lower it was in the frontal sites. Both morphometric and oscillatory patterns seem to reflect a lower engagement of frontal regulatory areas—relevant in sensitive caregiving—over the occipital areas in the NG. The lower fMRI pattern of activation in response to crying faces in occipital areas in the NG compared to the CG [6] may also reflect a lower top-down regulation from frontal regions. Mothers with neglectful behavior showed a streamlined reduction in the inferior fronto-temporo-occipital structural connectivity corresponding to the inferior longitudinal fasciculus (ILF) and inferior fronto-occipital fasciculus (IFOF) tracts interconnecting the face-responsive cortex and the limbic and frontal areas [24].

Notice that the cortical thickness reduction in the right RMFG and OFC in the NG versus CG can be related to these areas’ susceptibility to neurodegeneration [52], specifically related to age [53]. However, the influence of age can be ruled out here since the analysis was controlled for age effects and the mothers with neglectful behavior were significantly younger than the control mothers. Additionally, the frontal cortical thickness reductions observed in the NG in this study are in line with the substantial white matter volume reductions in bilateral frontal areas in the NG versus CG [7]. This convergence is congruent with studies reporting a certain correspondence between volumetric and cortical thickness measures in the target white matter regions [54]. Taken together, our results tentatively suggest that the lesser cortical thickness observed in the NG could be a result of accelerated/truncated pruning [55] and/or a concurrent loss of myelinated white matter that may have occurred during development [56]. Nevertheless, the direction of the relationship between CT and myelination is not yet clear, solely based on the morphometric measures.

The analysis of the cortical-EA associations showed direct effects of the right rostral middle frontal gyrus (RMFG) on alexithymia DIF and on the emotional availability exhibited in the mother-child interactive play task. The marginal indirect effect through alexithymia should not be totally ignored because it reduced the total relation of RMFG on EA, though this path remained significant. The RMFG-EA finding is in line with evidence showing that frontal areas are implicated in the bidirectional attunement of mother-child brain activity while watching animation videos together, suggesting their involvement in mother-child joint activity [57]. The right RMFG exerts control over attentional networks and is responsible for the flexible modulation of attention, orienting and reorienting attention from exogenous to endogenous attentional control [58]. This attention-control ability may allow for effective mother-child tuning during dyadic exchanges in joint activities. Our results are also in line with the relative sensitivity of cortical thickness features to adult differences in personality traits related to behavior [31]. The alexithymia factor corresponds to the difficulty in identifying feelings (DIF), which involves dysfunction in emotional awareness that is accompanied by a poor appreciation of own and others’ emotions, leading to ineffective emotional responses in the course of communicative dyadic exchanges [59]. Accordingly, greater cortical thickness in the RMFG and higher scores in the alexithymia DIF were, respectively, positively and negatively related to emotional availability. Of note, the factors of difficulty in identifying feelings and difficulty in verbalizing feelings, compared to the externally oriented thinking, are thought to reflect the affective aspects of alexithymia more related to emotional awareness [59].

The results of this study should be considered in light of two limitations. First, the small sample size prevents us from reaching definite conclusions concerning brain differences in cortical features, even though the clusters were identified in regions fitting with our hypotheses. Second, the cross-sectional design does not allow us to infer whether these cortical features have causal relationships with alexithymia and emotional availability.

## 5. Conclusions

This study brings preliminary novel evidence of distinctive cortical features in frontal and occipitotemporal cortices associated with maternal neglectful caregiving. In particular, the right rostral middle frontal gyrus, subserving control functions, and trait alexithymia, indexing a lower level of emotional awareness, were associated with the quality of mother-child interactions, providing new insights into the neural and personality correlates of these relationships. In sum, this study has advanced our understanding of the associations between brain structure and human mothering by identifying the distinctive cortical morphometric patterns associated with neglectful mothering and the poor mother-child bonding interactions.

## Figures and Tables

**Table 1:** Sociodemographic and neglect risk profile in Neglect and Control Groups. Group comparisons with mean scores were performed with *t* statistic, while those with percentage values were performed with chi-square (*χ*2) statistic.

	Neglect Group *n* = 24	Control Group *n* = 21	Comparisons *χ*2/*t*
*Sociodemographic profile*			
Mean age of the mother	29.1 (7.1)	33.6 (3.2)	−2.82 **
Number of children	2.08 (0.8)	1.57 (0.6)	2.24 *
Mean age of target child	2.7 (1.5)	2.3 (1.9)	0.82
Rural areas (%)	87.5	80.9	0.04
Level of education (%):			2.53
Primary	70	47.6	
Secondary	16.6	28.5	
>Secondary	12.5	23.8	
Single-parent family	50	14	4.92 *
Financial help from institutions	83	14	18.7 ***
*Neglect risk profile*			
History abuse/neglect (%)	67	14	10.5 **
Intimate partner conflict (%)	5	0	1.22
Chronic physical illness (%)	16	5	0.22
Poor household management (%)	88	0	24.4 ***
Disregard health/education needs (%)	61	0	12.4 ***
Disregard emotion/cognitive needs (%)	89	5	20.9 ***
Rigid/inconsistent norms (%)	67	5	11.4 ***
*Psychiatric Disorders factor*	0.67 (0.99)	−0.69 (0.23)	6.35 ***

* *p* < 0.05; ** *p* < 0.01; *** *p* < 0.001.

**Table 2:** Path analysis psychological variables. Alexithymia and Emotional Availability comparisons between Neglect and Control Groups.

	Neglect Group *n* = 24	Control Group *n* = 21	Comparisons	
	*M* (*SD*)	*M* (*SD*)	*T* (43)	*δ*
Alexithymia (averaged score)	3.10 (0.88)	2.45 (0.79)	2.56 *	0.77
Difficulty in Describing	3.35 (1.30)	2.59 (1.18)	2.03 *	0.60
Feelings				
Difficulty in Identifying	2.98 (1.31)	2.18 (1.07)	2.21 *	0.66
Feelings				
Externally Oriented Thinking	3.05 (0.61)	2.59 (0.56)	2.63 **	0.78
Emotional Availability (factor Score)	−0.62 (0.92)	0.71 (0.48)	−6.18 ***	1.78

* *p* < 0.05; ** *p* < 0.01; *** *p* < 0.001.

**Table 3:** Differential cortical features in Neglect versus Control groups. A cluster showing reduced cortical thickness for mothers of the neglect group (CG > NG) was found comprising the rostral middle frontal and orbitofrontal regions. The greater surface area was found in the lingual and lateral occipital gyri for the neglect group (CG < NG). Age, total intracranial volume for surface analyses only, and Psychiatric Disorders were included as nuisance covariates.

Cluster/Regions	Total Vertex	Cluster-Wise *p*-Value	Max x, y, z (mm)	Max −log10 (*p*-Value)
*Control Group > Neglect Group (Cortical thickness)*				
R. Rostral middle frontal, lateral and medial orbitofrontal	1434	0.014	21.1, 51.5, −12.5	3.45
*Control Group < Neglect Group (Surface area)*				
R. Lingual and lateral occipital	1185	0.0002	7.4, −88.3, −11.3	−3.35

## Data Availability

The structural MRI data and the covariates scores that support the findings of this study are available in a GIN repository: https://gin.g-node.org/lorna/data_surface_based_morphometry_study_neglectful_parenting, accessed on 20 December 2020, with the identifier DOI: 10.12751/g-node.6e3v37.

## Supplementary Materials

The following are available online at https://www.mdpi.com/2076-3425/11/3/387/s1, S1: Participant recruitment strategy and procedure; S2: Risk profile measures; Table S1: Psychopathological conditions stratified by Group; Table S2: Inter-rater reliabilities and on-factor component loadings of the Emotional Availability Scales; Table S3: Collinearity indexes between the Group and the psychopathological conditions.
